# Hybrid data fidelity term approach for quantitative susceptibility mapping

**DOI:** 10.1002/mrm.29218

**Published:** 2022-04-18

**Authors:** Mathias Lambert, Cristian Tejos, Christian Langkammer, Carlos Milovic

**Affiliations:** ^1^ Department of Electrical Engineering Pontificia Universidad Catolica de Chile Santiago Chile; ^2^ Biomedical Imaging Center Pontificia Universidad Catolica de Chile Santiago Chile; ^3^ Millennium Institute for Intelligent Healthcare Engineering (iHEALTH) Santiago Chile; ^4^ Department of Neurology Medical University of Graz Graz Austria; ^5^ BioTechMed Graz Graz Austria; ^6^ Department of Medical Physics and Biomedical Engineering University College London London UK

**Keywords:** Augmented Lagrangian, L1‐norm, L2‐norm, QSM, QSM challenge

## Abstract

**Purpose:**

Susceptibility maps are usually derived from local magnetic field estimations by minimizing a functional composed of a data consistency term and a regularization term. The data‐consistency term measures the difference between the desired solution and the measured data using typically the L2‐norm. It has been proposed to replace this L2‐norm with the L1‐norm, due to its robustness to outliers and reduction of streaking artifacts arising from highly noisy or strongly perturbed regions. However, in regions with high SNR, the L1‐norm yields a suboptimal denoising performance. In this work, we present a hybrid data fidelity approach that uses the L1‐norm and subsequently the L2‐norm to exploit the strengths of both norms.

**Methods:**

We developed a hybrid data fidelity term approach for QSM (HD‐QSM) based on linear susceptibility inversion methods, with total variation regularization. Each functional is solved with ADMM. The HD‐QSM approach is a two‐stage method that first finds a fast solution of the L1‐norm functional and then uses this solution to initialize the L2‐norm functional. In both norms we included spatially variable weights that improve the quality of the reconstructions.

**Results:**

The HD‐QSM approach produced good quantitative reconstructions in terms of structural definition, noise reduction, and avoiding streaking artifacts comparable with nonlinear methods, but with higher computational efficiency. Reconstructions performed with this method achieved first place at the lowest RMS error category in stage 1 of the 2019 QSM Reconstruction Challenge.

**Conclusions:**

The proposed method allows robust and accurate QSM reconstructions, obtaining superior performance to state‐of‐the‐art methods.

## 1 INTRODUCTION

Quantitative susceptibility mapping is an MRI reconstruction technique that allows the calculation of the magnetic susceptibility of tissues from the phase of gradient‐echo acquisitions.^1^ The magnetic susceptibility of a material is a property defined as the degree of magnetization of a material in the presence of an external magnetic field. Most biological brain tissues are intrinsically diamagnetic. Whereas diamagnetic myelin or calcium deposits are generating a magnetic field opposed to the applied field, paramagnetic materials, such as iron, react by generating a magnetic field in the same direction as that of the external field.^2^ Unlike conventional susceptibility‐sensitive techniques (eg, R_2_* mapping, susceptibility‐weighted imaging), QSM quantifies the diamagnetic and paramagnetic contributions, yielding exquisite contrast between anatomical structures.

Specific physiological and pathological processes change the magnetic susceptibility and QSM can be used to quantify oxygenation levels^3^ and detect hemorrhages and microhemorrhages.^4^ Increased regional susceptibilities have been consistently found in several neurodegenerative diseases,^2^ including Alzheimer's,^5^ Parkinson's,^6^, ^7^ Huntington's,^8^ and multiple sclerosis.^9^, ^10^

Susceptibility maps are typically calculated by following three consecutive processing steps: phase unwrapping,^11^ background field removal,^12^ and dipole inversion.^13^, ^14^, ^15^, ^16^, ^17^, ^18^, ^19^, ^20^ The unwrapping stage eliminates 2π jumps produced in the phase of the measured gradient‐echo signal. Background field removal eliminates the magnetic field contributions originated by objects outside the region of interest or FOV and field inhomogeneities, leaving only the magnetization field originated from local objects. The susceptibility‐to‐field model considers noninteracting magnetic dipoles, each associated with a single susceptibility source. This effectively models the measured magnetic field as the convolution between a dipole kernel and the underlying susceptibility distribution.^21^, ^22^ Therefore, the susceptibility distribution might be obtained by deconvolving the local magnetic fields by the dipole kernel. This process, known as the dipole inversion, is an ill‐posed inverse problem. The dipole kernel has a zero‐valued biconical surface in the Fourier domain, known as the “magic cone,” which impedes direct division. Truncated solutions^14^ (ie, replacing values below a threshold with a small number) amplify noise and contaminate the reconstructed susceptibility maps with streaking artifacts (ie, conical patterns originated from noisy voxels).

To address this issue, the dipole inversion process is usually reformulated as an optimization problem. Optimization models minimize a functional, usually composed of two terms: a data consistency and a regularization term. The regularization term is used to include prior information about the solution, which promotes desired characteristics, such as smoothness or continuous solutions (Tikhonov regularizer^16^) or piece‐wise constant solutions (total variation regularizer^23^). The data consistency—or data fidelity term—is a measure of the error between the proposed solution and the local magnetic fields, given the susceptibility‐to‐field or forward model.

Commonly, the data‐consistency term minimizes the squared difference between the dipole‐convolved solution and the local field (ie, a squared L2‐norm). The squared L2‐norm is a mathematically and computationally efficient function that also defines a convex penalty function that gives a unique solution. For QSM, this approach performs relatively well with data that have been corrupted by moderate amount of noise. However, the squared L2‐norm heavily penalizes large discrepancies produced by strong noise or other sources of discrepancy such as preprocessing artifacts. This high penalty tends to produce susceptibility maps with streaking artifacts, especially in low‐SNR areas. This behavior might be explained from a Bayesian point of view. Finding the solution that minimizes the L2‐norm with noise‐corrupted measured data is equivalent to finding the maximum likelihood estimate in a maximum a posteriori probability problem, but this only happens when the noise source has a Gaussian distribution. Indeed, whereas the noise distribution in phase MRI signals with high SNR can be approximated as Gaussian, this approximation is no longer valid for low SNR.^24^, ^25^, ^26^

To address this problem, Liu et al^17^ proposed a nonlinear data fidelity term that computes the error of the forward model at the complex image domain, improving robustness to noise at the expense of higher computational cost. Later, Milovic et al^20^ proposed using an L1‐norm data‐consistency term (least absolute error minimization), producing a better performance against outlier voxels.^27^ Compared with the L2‐norm, the L1‐norm penalizes large discrepancies between the proposed solution and the measured data less severely. This prevents energy spilling from voxels with large discrepancies with respect to their neighbors, which in turn reduces the generation and propagation of streaking artifacts. However, from a Bayesian point of view, the L1‐norm does not have similar denoising capabilities as the L2‐norm, thus the resulting images have a residual noise component and lower SNR.^20^

In this paper we present a hybrid data fidelity term approach for QSM (HD‐QSM). This dipole inversion algorithm sequentially uses linear L1‐norms and L2‐norms for data consistency. The resulting algorithm successfully combines the strengths of both norms. The HD‐QSM approach participated at the 2019 QSM Reconstruction Challenge (RC2, Seoul, Korea),^28^, ^29^ obtaining the first place at the lowest RMS error category in stage 1. We here present a full validation of our previously reported method^30^ using simulations and in vivo data, and exhaustive comparisons with alternative methods.

## 2 METHODS

### 2.1 Hybrid data fidelity term approach for QSM

The proposed method HD‐QSM consists of two stages. The first stage finds a suitable initial solution that is robust to streaking artifacts.^20^ For this purpose, we use the following linear optimization problem:

(1) $$\chi_{1}=\underset{\chi }{\text{argmin}}{\left\|w\left(F^{H}\mathrm{DF}\chi -\phi \right)\right\|}_{1}+\lambda_{1}^{L_{1}}\cdot \mathrm{TV}\left(\chi \right),$$

where ${\left\|\cdot \right\|}_{1}$ is the L1‐norm; F is the Fourier transform with its inverse $F^{H}$; $D=\gamma H_{0}\mathrm{TE}\left(\frac{1}{3}-\frac{k_{z}^{2}}{k^{2}}\right)$ is the dipole kernel; ϕ is the local phase map; $\chi_{1}$ is the susceptibility distribution obtained in this first stage; TV(·) is the total variation regularizer^23^; $\lambda_{1}^{L_{1}}$ is the regularization weight used in the first stage. The value of w is a region‐of‐interest binary mask or a magnitude‐based weight, as follows:

(2) $$w=\frac{\sum_{i=1}^{N}\mathrm{Ma}g_{i}^{2}\cdot TE_{i}}{\sum_{i=1}^{N}\mathrm{Ma}g_{i}\cdot TE_{i}},$$

where N is the number of echoes; $\mathrm{Ma}g_{i}$ is the magnitude of echo i; and $TE_{i}$ is the *i*th TE. The idea behind the weight is to improve the SNR to equal that of the local field, using magnitude‐weighted least‐squares magnitude fitting similar to echo combination.

The second stage solves the following linear functional, using the solution of the previous stage ($\chi_{1}$) as the initialization:

(3) $$\underset{\chi }{\chi_{2}=\text{argmin}}\frac{1}{2}{\left\|W\left(F^{H}\mathrm{DF}\chi -\phi \right)\right\|}_{2}^{2}+\lambda_{1}^{L_{2}}\cdot \mathrm{TV}\left(\chi \right),$$

where ${\left\|\cdot \right\|}_{2}$ is the L2‐norm. The value of W is a spatially variable weight modulated by the voxel‐wise phase discrepancy factor between the solution obtained in the first stage (convolved by the dipole kernel) and the acquired local phase, as follows:

(4) $$W=w\cdot \left(1-\frac{\left|\phi -F^{H}\mathrm{DF}\chi_{1}\right|}{\max\left(\left|\phi -F^{H}\mathrm{DF}\chi_{1}\right|\right)}\right).$$

The discrepancy factor prevents the propagation of streaking artifacts by enforcing the data‐consistency term to penalize areas with low SNR and areas contaminated with phase inconsistencies less heavily.

The functionals were solved using the ADMM framework^19^ as described for FANSI^18^ and L1‐QSM.^20^ A straightforward implementation would require one to fine‐tune six parameters: two regularization weights $\left(\lambda_{1}^{L_{1}},,,\lambda_{1}^{L_{2}}\right)$ and four Lagrangian weights derived from the ADMM solver associated with gradient consistency weights $\left(\mu_{1}^{L1},,,\mu_{1}^{L2}\right)$ and data fidelity consistency weights $\left(\mu_{2}^{L1},,,\mu_{2}^{L2}\right)$. These Lagrangian weights are introduced by the variable splitting procedure of ADMM, as described in the Supporting Information. As in most optimization‐based QSM algorithms, parameters must be set using some heuristics^31^ (eg, L‐curve approach^32^). To simplify the parameter‐tuning process, we propose the following heuristic, derived from the numerical relationship between the L1 and L2 norms: $\lambda_{1}^{L_{1}}=\sqrt{\lambda_{1}^{L_{2}}},\mu_{1}^{L1}=\sqrt{\mu_{1}^{L2}},\mu_{2}^{L1}=\mu_{2}^{L2}=1$. Considering also the heuristic proposed for FANSI,^18^ $10\leq \frac{\mu_{1}}{\lambda_{1}}\leq 100$, where values within the range do not produce major variations on the optimal reconstruction. We therefore simplify the parameter‐setting problem to a one free parameter, $\lambda_{1}^{L_{2}}$.

The number of iterations in each stage $\left(i_{1},,,i_{2}\right)$ can be considered as free parameters to be tuned. However, as shown in our experiments, these are not sensitive parameters and might be fixed a priori. Considering a total number of iterations N, we recommend $i_{1}\in \left[10,100\right]$ and $i_{2}=N-i_{1}$. For the RC2, our winning reconstructions used this heuristic with the following parameters: $i_{1}=20$, $i_{2}=280$, $\lambda_{1}^{L_{2}}=6.3096\times 10^{-6}$, and $\frac{\mu_{1}}{\lambda_{1}}=10$.

The HD‐QSM approach was compared with alternative linear and nonlinear QSM methods using total variation as the regularizer. For convenience, we will use the following nomenclature: L1 and nlL1 correspond to the linear and nonlinear L1‐norm methods proposed in L1‐QSM,^20^ respectively; L2 and nlL2 correspond to the linear and nonlinear L2‐norm methods included in the FANSI Toolbox^18^ (ADMM‐based fast solvers for the total variation–regularized cost functions, similar to linear and nonlinear MEDI^17^), respectively; L1L2 corresponds to the HD‐QSM method with the proposed heuristic; and L1L2wH corresponds to the HD‐QSM without the heuristic (ie, optimizing each parameter independently). Comparisons were performed using synthetic data and in vivo acquisitions, as described subsequently.

## 3 EXPERIMENTAL DESIGN

### 3.1 COSMOS forward simulation

We used the COSMOS (calculation of susceptibility through multiple orientation sampling)^33^ reconstruction included in the 2016 QSM Reconstruction Challenge^34^ data set as ground truth. We forward‐simulated the phase and added complex Gaussian noise with SNR = 40, 100, and 300. Additionally, we forward‐simulated the phase (SNR = 100) with two phase jumps (±20π) to generate strong phase inconsistencies. These phase simulations were used as input for the QSM reconstructions of L1, L2, and L1L2 methods to compare their performances. Optimal reconstructions were obtained for each method by optimizing the normalized RMS error (NRMSE). We performed a sensitivity analysis evaluating the quality of the reconstructions around the optimal regularization parameter $\left(\lambda^{*}\right)$ within a range defined by $\left[0.1\cdot \lambda^{*},,,10\cdot \lambda^{*}\right]$, sampled at $\lambda_{i}=\lambda^{*}\cdot 10^{\frac{i}{30}}$, with i=[−30,−29,…,29,30].

### 3.2 2019 QSM challenge—SNR1 data set

In the context of the 2019 QSM challenge,^28^ two simulated data sets^29^ with different SNRs were provided. Each data set consisted of two brain images: Sim1 and Sim2. Sim2 had higher contrast between white matter and gray matter than Sim1. Additionally, a strong calcification was included in Sim2. We used the SNR1 data set, as it presents a lower SNR ratio (SNR1 = 100 vs SNR2 = 1000). We estimated the local magnetic field from the phase of the simulated multi‐echo acquisitions using a magnitude‐weighted least‐squares fitting. Field maps were zero‐padded to 256 × 256 × 256 to prevent large‐scale aliasing and other artifacts. All reconstructions were stopped when they reached 300 iterations, and the reconstruction parameters were optimized to minimize NRMSE. For each optimal NRMSE reconstructions, we computed the error metrics used in RC2, namely^28^ dNRMSE, dNRMSE TISSUE, dNRMSE DeepGM, dNRMSE blood, calcification streaking, and deviation from calcification moment. We considered two additional global metrics: susceptibility‐tuned SSIM^35^ and the high‐frequency error norm.^36^

### 3.3 In vivo data set

We performed an in vivo acquisition on a Siemens 3T scanner (Magnetom Trio Tim; Siemens Healthcare, Erlangen, Germany) with a 12‐channel phased‐array head coil. We used a gradient‐echo sequence with six echoes of a patient showing extensive brain hemorrhage with the following sequence parameters: TE_1_ = 4.92 ms, ΔTE = 4.92 ms, TR = 35 ms, flip angle = 15°, matrix = 232 × 288 × 64 with 0.8 × 0.8 × 2 mm^3^ voxel size, and acquisition time = 4:51 min. Phase unwrapping was performed with SEGUE (speedy region‐growing algorithm for unwrapping estimated phase),^37^ and background field removal was performed by projection onto dipole fields.^38^ We estimated the local field using a magnitude‐weighted least‐squares phase fitting. Background field residuals were removed using the harmonic phase estimation obtained with the weak‐harmonic QSM method.^39^ Two additional experiments with in vivo data sets are included in Supporting Information Section E.

MR images from patients with brain hemorrhage were selected retrospectively. The study was approved by the IRB and the subjects gave informed consent.

## 4 RESULTS

### 4.1 COSMOS forward simulation

**FIGURE 1:**
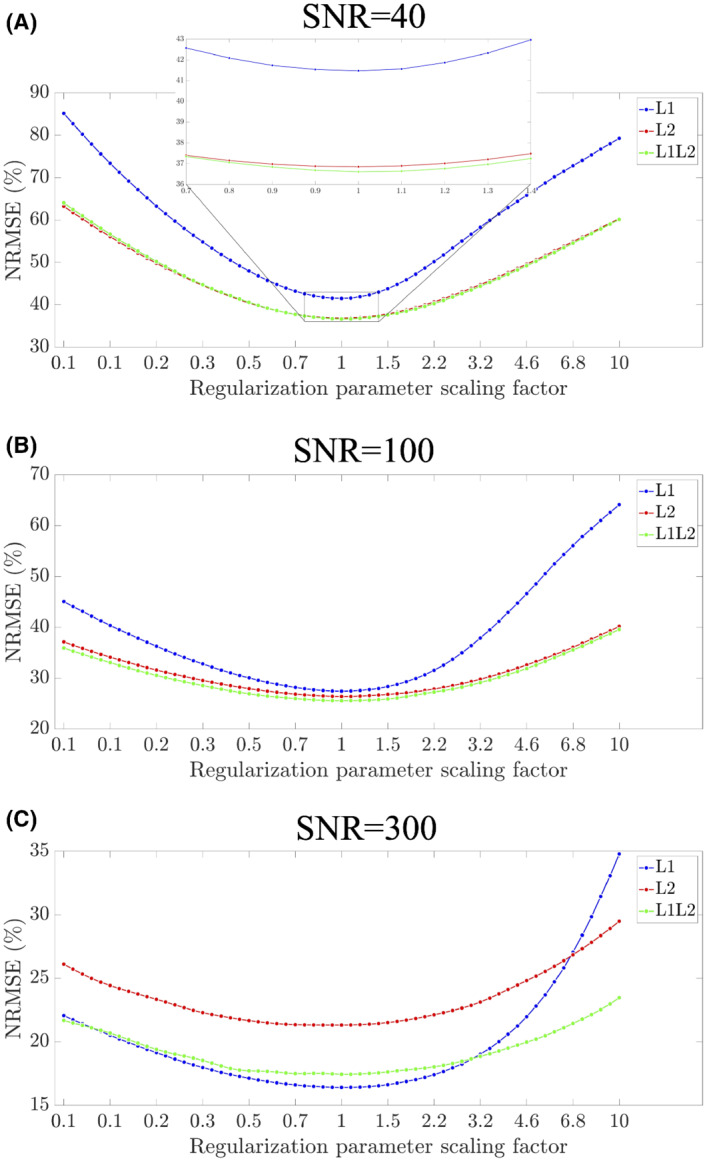
(A–C) The RMS error for different regularization parameters normalizing the scale with center at the optimum. Abbreviation: NRMSE, normalized RMS error

The L1L2 reconstruction obtained the best performance for medium and low SNRs (Figure 1A,B), whereas L1 achieved the best results for high SNR (Figure 1C). Independent of the SNR level, L1L2 obtained the most stable performance, by varying the regularization parameter around the optimum produced the smallest NRMSE change. The same performance was obtained by analyzing 11 zones of interest (Supporting Information Table S1), as described in Langkammer et al.^34^

**FIGURE 2:**
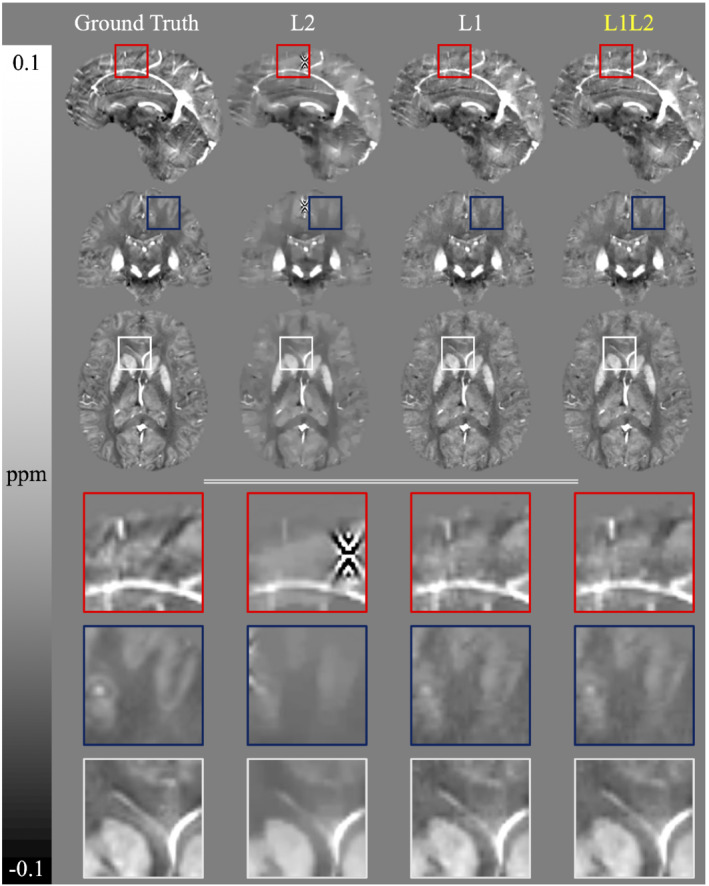
Optimal reconstructions of L1, L2, and L1L2 over the simulation with SNR = 100 and phase jumps. Areas of interest are enclosed in bounding boxes and magnified to show details

Although L2 reconstructions yielded streaking artifacts in the presence of phase inconsistencies, both L1 and L1L2 could successfully suppress those artifacts (Figure 2). To compensate for the generation of streaking artifacts, minimization of the NRMSE produced an overregularized L2 reconstruction. The L1L2 reconstructions demonstrated better delineation or definition (without overregularization) of small blood vessels, along with better contrast between white matter and gray matter.

### 4.2 QSM challenge 2.0—SNR1 data set

The HD‐QSM (L1L2 and L1L2wH) reconstruction achieved the best performance in most of the analyzed metrics, especially when considering RMS error–based metrics (Table 1). The metrics obtained using the proposed heuristic (L1L2) are similar to those obtained by tuning all parameters (L1L2wH). The nlL1 reconstruction obtained the second‐best performance in NRMSE (optimized variable), but the processing time was more than 2 times larger than those of the linear competitors.

**TABLE 1:**
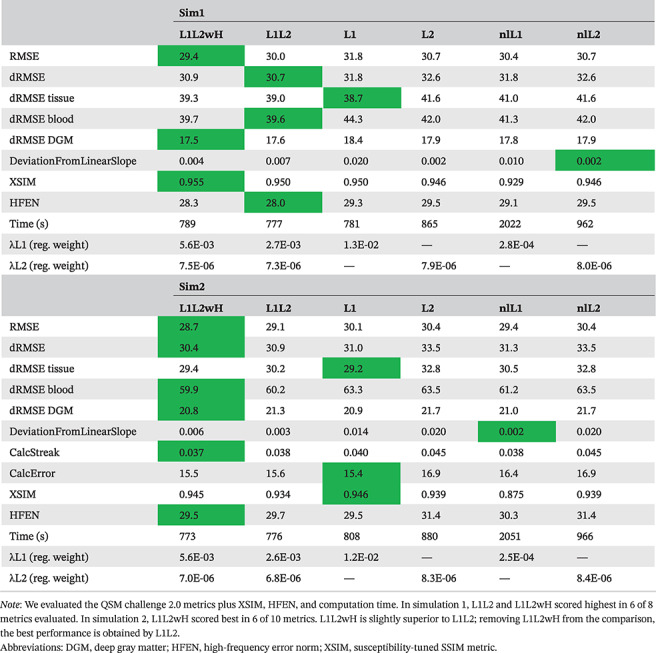
Metrics of RMSE‐based optimal reconstructions

The optimal number of L1 iterations ($i_{1}$) was 40 and 280 for L1L2 and L1L2wH, respectively. For different $i_{1}$, the optimized reconstructions of L1L2 and L1L2wH showed differences less than 1% in NRMSE (Supporting Information Figures S1–S5).

**FIGURE 3:**
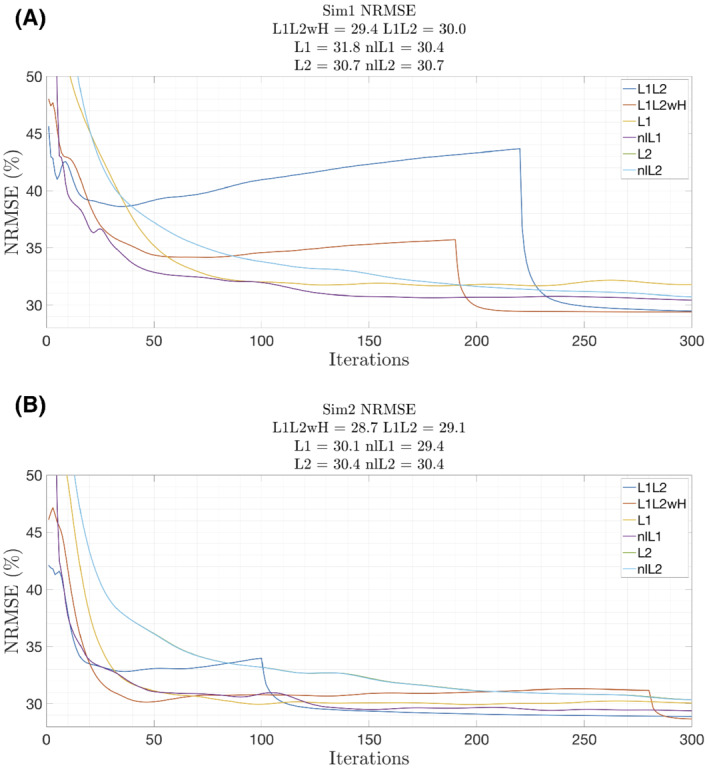
Evolution of NRMSE by iterations for all methods on Sim1 and Sim2. The curves of L2 and nlL2 overlap. The first stage of L1L2 and L1L2wH diverges, while stage 2 converges fast. The difference in NRMSE between L1L2 and L1L2wH is 0.5 points

The reconstructions obtained for each method are presented in Supporting Information Figures S6 and S7. Figure 3 shows the evolution of NRMSE per iteration of the optimal reconstructions achieved for each method. The curves for L2 and nlL2 are overlapped, as their performances were almost identical. Stage 1 of L1L2 and L1L2wH diverged before the transition to stage 2, and then it quickly converged again.

The optimal regularization weights of our proposed methods (L1L2 and L1L2wH) were smaller than those respectively obtained for the L1 and L2 methods (Table 1). Having a smaller $\lambda_{1}$ might explain the divergence observed in NRMSE curves in our stage 1. Supporting Information Figures S4 and S5 present reconstructions at the end of stage 1 and stage 2, with and without the use of the weight modulated by the voxel‐wise phase discrepancy factor. Use of the discrepancy factor helps the L2‐norm to reduce the artifacts present in the image provided by stage 1. This is done by penalizing with a low weight those voxels that might produce strong artifacts.

### 4.3 In vivo data set

**FIGURE 4:**
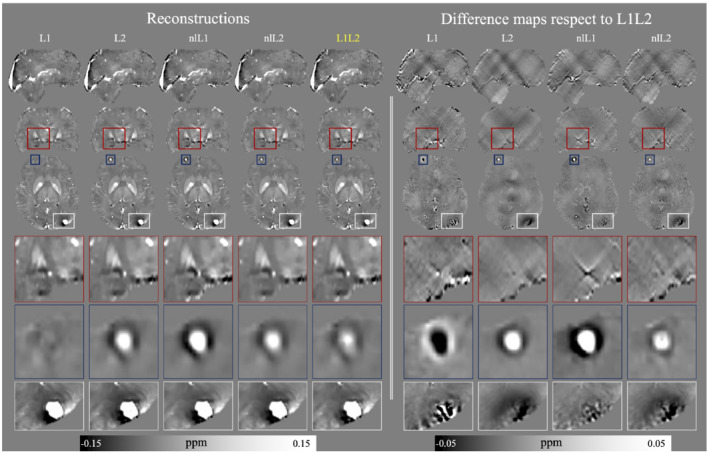
Optimal reconstructions obtained by visual inspection around the optimum indicated by the L‐curve analysis. Areas of interest are enclosed in bounding boxes. The red box shows the basal ganglia region and encloses a zone with a hyperintense structure (might be a blood vessel shown as a white circle) in the center. nlL1 generates an artifact in the structure, generating a different geometry and propagating a streaking artifact. The blue and white boxes enclose frontal and posterior lesions, respectively. L1L2 generates reconstructions with less artifacts around the lesions

Figure 4 presents the in vivo reconstructions for each method. The optimal reconstructions were chosen by visual inspection around the optimum indicated by the L‐curve analysis. The reconstruction of the linear L1‐norm method shows a hallucinated suppression of the frontal lesion, which limits the clinical useability of this method. All other methods were able to successfully recover the lesions, with L1L2 showing the fewest shadow artifacts adjacent to the frontal and posterior lesions.

The difference maps highlight that the L1L2 reconstruction is smooth like the L2‐norm reconstructions, but comes with a better structural definition such as the nlL1 reconstruction. The L1L2 and nlL1 reconstructions resolved structural details such as the posterior lesion, generating only minor shadows around it.

Supporting Information Figures S8 and S9 present additional in vivo data reconstructions. In these less‐challenging data sets, the proposed L1L2 method better reconstructs structures such as the vein shown in Supporting Information Figure S8 and mitigates the streaking artifact shown in Supporting Information Figure S9.

## 5 DISCUSSION

The hybrid data fidelity term approach for QSM is an iterative method that differs from current methods in that it is composed of two consecutive stages using the L1 and L2 norm in a linear data consistency term. The HD‐QSM method provides good reconstructions at low, medium, and high SNRs, and obtains superior performances at low and medium SNRs compared with single‐stage linear methods using the L1‐norm, or alternatively, the L2‐norm. The use of two stages allows HD‐QSM to exploit the strengths of both norms. The reconstructions are robust to outliers, providing good noise‐reduction capabilities and stability with respect to the regularization parameter.

The data‐consistency weights (Eqs. 2 and 4) play a fundamental role in HD‐QSM. They help to identify voxels that generate artifacts so that the L2‐norm data consistency penalizes them less heavily and avoids generating artifacts. It also allows stage 1 to search for a subregularized solution rich in structural information and provides stage 2 with an initial solution closer to the optimum. This makes HD‐QSM regularization parameters lower than their single‐stage counterparts. Similarly to the MERIT^17^ heuristic, the L1‐norm rejects outliers dynamically at each iteration, but at a threshold that depends on the data distribution without the need of parameter tuning. The redefined data‐consistency weight (Eq. 4) maintains the effect of this threshold on the last iteration of stage 1 throughout stage 2.

Our method has six free parameters to be tuned. However, we propose a heuristic to reduce this complexity to only one free parameter. After a fixed total number of iterations, the difference of the NRMSE obtained between the six free parameter method and the proposed heuristic was always less than 1%, independent of the distribution of iterations between the first and the second step. Even though the L1 minimization step appears to diverge after a few iterations, the L2 stage plus the use of data‐consistency weights can rapidly reduce the errors. In other words, setting only one free parameter and running only a few iterations for the L1 minimization step would be enough to define a good initialization for the L2 minimization step, and lately, achieve high‐quality QSM reconstructions.

In terms of image quality, the proposed two‐stage solver represents an improvement over linear and nonlinear formulations in terms of reducing noise and preventing streaking artifacts emanating from low‐SNR regions. Compared with L2‐norm methods, HD‐QSM produced reconstructions with better structural definition and better artifact management. Compared with L1‐norm methods, HD‐QSM produced reconstructions with a less noisy visual appearance and closer to the ground truth. The HD‐QSM method requires similar computational time compared with linear methods and outperforms nonlinear methods.

The idea of solving QSM reconstructions using a previous reconstruction as a starting point might be extended to single‐step formulations (ie, including phase unwrapping and background field removal into the functionals). Initialization based on solutions that do not require parameter tuning can also be explored (ie, nonregularized functional, deep learning models), in which case this model would serve as a refinement step.

## 6 CONCLUSIONS

The HD‐QSM method combines the beneficial features of the L1‐norm and L2‐norm to obtain high‐quality QSM reconstructions (ie, good structural definition, noise reduction and preventing streaking artifacts), while maintaining the computational complexity of a linear method. We also proposed a simple and effective heuristic that reduces fine‐tuning to only one parameter to achieve optimal performance. The HD‐QSM method demonstrated exquisite numerical performance in the QSM challenge 2.0 and in pathological MRI data sets with structural abnormalities and conspicuous features.

## Supporting information


**Table S1.** Local measurements (mean value ± SD [local RMSE], in parts per billion) of evaluation areas for the COSMOS (calculation of susceptibility through multiple orientation sampling)–based phantom
**Figure S1.** Normalized RMS error (NRMSE) evolution of Sim1 optimal reconstructions for different $i_{1}$. The error difference obtained between the free parameter method and the proposed heuristic is less than 1 point for all $i_{1}$. The difference between the best and the worst reconstruction is less than 1 point, indicating that the number of iterations of L1‐norm is not an extremely determinant factor, which confirms the hypothesis that the stage of an L1‐norm solution is a better starting point than 0
**Figure S2.** The NRMSE evolution of Sim2 optimal reconstructions for different $i_{1}$
**Figure S3.** Solutions at the end of stage 1 and stage 2 for Sim1 and Sim2. The solutions at the end of stage 1 show a noisy appearance with streaking artifacts (see around the calcification), but with good structural definition. The final solutions maintain the structural details but do not show the noise and streaking artifacts
**Figure S4.** The first column presents the solution at the end of stage 1; the second column shows the discrepancy factor, which weights the data‐consistency weight; the third column shows the solution of stage 2 using the adjustment factor; and the fourth column shows the solution without using the adjustment factor with the same parameters
**Figure S5.** The NRMSE‐optimized solutions of the hybrid data fidelity term approach for QSM (HD‐QSM) without the discrepancy factor. The first column presents the solution at the end of stage 1, and the second column shows the final solution
**Figure S6.** Optimal NRMSE reconstructions of Sim 1. For the search of the optimum of L1L2wH, a search was performed in a vector space of 5 × 5 × 5 × 5 $\left(\lambda_{1}^{L_{1}},,,\mu_{1}^{L_{1}},,,\lambda_{1}^{L_{2}},,,\mu_{1}^{L_{2}}\right)$; once the optimum of this space was located, a second search was performed in a space of the same size in the vicinity of the optimum. In total, three search processes were performed for each simulation, which equates to 3750 reconstructions, whereas for L1L2 only 50 reconstructions were necessary
**Figure S7.** Optimal NRMSE reconstructions of Sim2. To find the optimum of L1L2wH, a search was performed in a vector space of 5 × 5 × 5 × 5 $\left(\lambda_{1}^{L_{1}},,,\mu_{1}^{L_{1}},,,\lambda_{1}^{L_{2}},,,\mu_{1}^{L_{2}}\right)$. Once the optimum of this space was located, a second search was performed in a space of the same size in the vicinity of the optimum. A total of three search processes were performed for each simulation, which equates to 3750 reconstructions, whereas for L1L2, only 50 reconstructions were necessary
**Figure S8.** Additional in vivo reconstruction of a healthy patient. The red arrows indicate a cortical vein. L1L2 and nlL1 were able to correctly reconstruct the veins of the cortex
**Figure S9.** Additional in vivo reconstruction of a patient with brain calcifications. Red arrows indicate the origin of a streaking artifact. The L1L2 reconstruction succeeds in mitigating this spread.

## Data Availability

The source code from this paper is openly available in https://github.com/mglambert/HDQSM.
