# Supplementary material for: Hybrid data fidelity term approach for quantitative susceptibility mapping
Source: Magn Reson Med. 2022 Apr 18;88(2):962–72. doi: 10.1002/mrm.29218 (PMC9324845; doi:10.1002/mrm.29218)
Supplement: Supplementary file 1 — Table S1. Local measurements (mean value ± SD [local RMSE], in parts per billion) of evaluation areas for the COSMOS (calculation of susceptibility through multiple orientation sampling)–based phantom Figure S1. Normalized RMS error (NRMSE) evolution of Sim1 optimal reconstructions for different i1. The error difference obtained between the free parameter method and the proposed heuristic is less than 1 point for all i1. The difference between the best and the worst reconstruction is less than 1 point, indicating that the number of iterations of L1‐norm is not an extremely determinant factor, which confirms the hypothesis that the stage of an L1‐norm solution is a better starting point than 0 Figure S2. The NRMSE evolution of Sim2 optimal reconstructions for different i1 Figure S3. Solutions at the end of stage 1 and stage 2 for Sim1 and Sim2. The solutions at the end of stage 1 show a noisy appearance with streaking artifacts (see around the calcification), but with good structural definition. The final solutions maintain the structural details but do not show the noise and streaking artifacts Figure S4. The first column presents the solution at the end of stage 1; the second column shows the discrepancy factor, which weights the data‐consistency weight; the third column shows the solution of stage 2 using the adjustment factor; and the fourth column shows the solution without using the adjustment factor with the same parameters Figure S5. The NRMSE‐optimized solutions of the hybrid data fidelity term approach for QSM (HD‐QSM) without the discrepancy factor. The first column presents the solution at the end of stage 1, and the second column shows the final solution Figure S6. Optimal NRMSE reconstructions of Sim 1. For the search of the optimum of L1L2wH, a search was performed in a vector space of 5 × 5 × 5 × 5 λ1L1,μ1L1,λ1L2,μ1L2; once the optimum of this space was located, a second search was performed in a space of the same size in the vicinity of the optimum. [file MRM-88-962-s001.pdf]

Hybrid data fidelity term approach for Quantitative Susceptibility Mapping

Mathias Lambert, Cristian Tejos, Christian Langkammer, Carlos Milovic.

Support Information

Section A - ADMM Solver for Stage 1

The first stage of HD-QSM is to solve the following linear QSM functional with L1-norm:

$$\min_{\chi_1} \left\| w \cdot \left(F^H D F \chi_1 - \phi \right) \right\|_1 + \lambda_1^{L_1} \left\| \nabla \chi_1 \right\|_1 \tag{S.1}$$

Using ADMM, we introduced an auxiliary variable $z_1 = \nabla \chi_1$, and decoupled the equation system, leading to the following augmented Lagrangian functional:

$$\min_{\chi_1, z_1} \left\| w \cdot \left(F^H D F \chi_1 - \phi \right) \right\|_1 + \lambda_1^{L_1} \left\| z_1 \right\|_1 + \frac{\mu_1^{L_1}}{2} \left\| \nabla \chi_1 - z_1 + s_1 \right\|_2^2 \tag{S.2}$$

where s_1 is an Lagrange multiplier and $\mu_1^{L_1} > 0$ is a penalty parameter, in this case called gradient consistency weight. To decouple the χ_1 subproblem we introduced $z_2 = F^H D F \chi_1 - \phi$:

$$\min_{\chi_1, z_1, z_2} \left\| w \cdot z_2 \right\|_1 + \lambda_1^{L_1} \left\| z_1 \right\|_1 + \frac{\mu_1^{L_1}}{2} \left\| \nabla \chi_1 - z_1 + s_1 \right\|_2^2 + \frac{\mu_2^{L_1}}{2} \left\| F^H D F \chi_1 - \phi - z_2 + s_2 \right\|_2^2 \tag{S.3}$$

where s_2 is an Lagrange multiplier and $\mu_2^{L_1} > 0$ is called data fidelity consistency weight.
We solved the χ_1 subproblem, the gradient operator can be decomposed as $\nabla = F^H E F$, where E is a diagonal matrix that represents the differential operation in frequency domain:

$$\min_{\chi_1} \frac{\mu_1^{L_1}}{2} \left\| F^H E F \chi_1 - z_1 + s_1 \right\|_2^2 + \frac{\mu_2^{L_1}}{2} \left\| F^H D F \chi_1 - \phi - z_2 + s_2 \right\|_2^2 \tag{S.4}$$

$$\frac{\partial}{\partial \chi_1} \left(\frac{\mu_1^{L_1}}{2} \left\| F^H E F \chi_1 - z_1 + s_1 \right\|_2^2 + \frac{\mu_2^{L_1}}{2} \left\| F^H D F \chi_1 - \phi - z_2 + s_2 \right\|_2^2 \right) = 0 \tag{S.5}$$

$$\mu_1^{L_1} F^H E^H F \left(F^H E F \chi_1 - z_1 + s_1 \right) + \mu_2^{L_1} F^H D^H F \left(F^H D F \chi_1 - \phi - z_2 + s_2 \right) = 0 \tag{S.6}$$

$$\boxed{\chi_1 = F^H \frac{\mu_1^{L_1} E^H F \left(z_1 - s_1 \right) + \mu_2^{L_1} D^H F \left(z_2 - s_2 + \phi \right)}{\mu_1^{L_1} E^H E + \mu_2^{L_1} D^H D}} \tag{S.7}$$

We solved the z_1 subproblem by the soft thresholding operation:

$$\min_{z_1} \lambda_1^{L_1} \left\| z_1 \right\|_1 + \frac{\mu_1^{L_1}}{2} \left\| \nabla \chi_1 - z_1 + s_1 \right\|_2^2 \tag{S.8}$$

$$\frac{\partial}{\partial z_1} \left(\lambda_1^{L_1} \left\| z_1 \right\|_1 + \frac{\mu_1^{L_1}}{2} \left\| \nabla \chi_1 - z_1 + s_1 \right\|_2^2 \right) = 0 \tag{S.9}$$

$$\lambda_1^{L_1} \operatorname{sign}(z_1) - \mu_1^{L_1} \left(\nabla \chi_1 - z_1 + s_1 \right) = 0 \tag{S.10}$$

In the optimal $z_1 = (\nabla \chi_1 + s_1) - \frac{\lambda_1^{L_1}}{\mu_1^{L_1}} \operatorname{sign}(z_1)$. If $z_1 < 0 \implies (\nabla \chi_1 + s_1) < \frac{\lambda_1^{L_1}}{\mu_1^{L_1}} \operatorname{sign}(z_1)$ and equivalently if $z_1 > 0 \implies (\nabla \chi_1 + s_1) > \frac{\lambda_1^{L_1}}{\mu_1^{L_1}} \operatorname{sign}(z_1)$. Thus $|\nabla \chi_1 + s_1| > \frac{\lambda_1^{L_1}}{\mu_1^{L_1}} \implies \operatorname{sign}(\nabla \chi_1 + s_1) = \operatorname{sign}(z_1)$. Then:

$$z_1 = (\nabla \chi_1 + s_1) - \frac{\lambda_1^{L_1}}{\mu_1^{L_1}} \operatorname{sign}(\nabla \chi_1 + s_1)$$

In the case $z_1 = 0 \implies \operatorname{sign}(\nabla\chi_1 + s_1) \in [-1, 1]$ the optimal condition is: $0 = (\nabla\chi_1 + s_1) - \frac{\lambda_1^{L_1}}{\mu_1^{L_1}}\operatorname{sign}(\nabla\chi_1 + s_1) \iff (\nabla\chi_1 + s_1) \in \left[-\frac{\lambda_1^{L_1}}{\mu_1^{L_1}}, \frac{\lambda_1^{L_1}}{\mu_1^{L_1}}\right] \iff |\nabla\chi_1 + s_1| \leq \left|\frac{\lambda_1^{L_1}}{\mu_1^{L_1}}\right|$. Thus

$$\boxed{z_1 = \text{máx}\left(|\nabla\chi_1 + s_1| - \frac{\lambda_1^{L_1}}{\mu_1^{L_1}}, 0\right) \cdot \operatorname{sign}(\nabla\chi_1 + s_1)} \tag{S.11}$$

We solved the z_2 subproblem by the soft thresholding operation as well:

$$\text{mín}_{z_2} \|w \cdot z_2\|_1 + \frac{\mu_2^{L_1}}{2}\left\|F^H DF\chi_1 - \phi - z_2 + s_2\right\|_2^2 \tag{S.12}$$

$$\boxed{z_2 = \text{máx}\left(\left|F^H DF\chi_1 - \phi + s_2\right| - \frac{w}{\mu_2^{L_1}}, 0\right) \cdot \operatorname{sign}\left(F^H DF\chi_1 - \phi + s_2\right)} \tag{S.13}$$

The update rules for the Lagrangian multipliers are given by:

$$s_1 = s_1 + F^H EF\chi_1 - z_1 \tag{S.14}$$

$$s_2 = s_2 + F^H DF\chi_1 - \phi - z_2 \tag{S.15}$$

Section B - ADMM Solver for Stage 2

The second stage of HD-QSM is to solve the following linear QSM functional with L2-norm:

$$\min_{\chi_2} \frac{1}{2} \left\| \mathbb{W} \cdot \left(F^H D F \chi_2 - \phi \right) \right\|_2^2 + \lambda_1^{L_2} \left\| \nabla \chi_2 \right\|_1 \tag{S.16}$$

where

$$\mathbb{W} \quad = \quad w \cdot \left(1 - \frac{\left| \phi - F^H D F \chi_1 \right|}{\max \left(\left| \phi - F^H D F \chi_1 \right| \right)} \right) \tag{S.17}$$

Using ADMM, we introduced an auxiliary variable $z_1 = \nabla \chi_2$, and decoupled the equation system, leading to the following augmented Lagrangian functional:

$$\min_{\chi_2, z_1} \frac{1}{2} \left\| \mathbb{W} \cdot \left(F^H D F \chi_2 - \phi \right) \right\|_2^2 + \lambda_1^{L_2} \left\| z_1 \right\|_1 + \frac{\mu_1^{L_2}}{2} \left\| \nabla \chi_2 - z_1 + s_1 \right\|_2^2 \tag{S.18}$$

To decouple the χ_2 subproblem we introduced $z_2 = F^H D F \chi_2 - \phi$:

$$\min_{\chi_2, z_1, z_2} \left\| \mathbb{W} \cdot z_2 \right\|_2^2 + \lambda_1^{L_2} \left\| z_1 \right\|_1 + \frac{\mu_1^{L_2}}{2} \left\| \nabla \chi_2 - z_1 + s_1 \right\|_2^2 + \frac{\mu_2^{L_2}}{2} \left\| F^H D F \chi_2 - \phi - z_2 + s_2 \right\|_2^2 \tag{S.19}$$

We solved the χ_2 subproblem by closed form:

$$\min_{\chi_2} \frac{\mu_1^{L_2}}{2} \left\| F^H E F \chi_2 - z_1 + s_1 \right\|_2^2 + \frac{\mu_2^{L_2}}{2} \left\| F^H D F \chi_2 - \phi - z_2 + s_2 \right\|_2^2 \tag{S.20}$$

$$\frac{\partial}{\partial \chi_1} \left(\frac{\mu_1^{L_2}}{2} \left\| F^H E F \chi_2 - z_1 + s_1 \right\|_2^2 + \frac{\mu_2^{L_2}}{2} \left\| F^H D F \chi_2 - \phi - z_2 + s_2 \right\|_2^2 \right) = 0 \tag{S.21}$$

$$\mu_1^{L_2} F^H E^H F \left(F^H E F \chi_2 - z_1 + s_1 \right) + \mu_2^{L_2} F^H D^H F \left(F^H D F \chi_2 - \phi - z_2 + s_2 \right) = 0 \tag{S.22}$$

$$\boxed{\chi_2 = F^H \frac{\mu_1^{L_2} E^H F \left(z_1 - s_1 \right) + \mu_2^{L_2} D^H F \left(z_2 - s_2 + \phi \right)}{\mu_1^{L_2} E^H E + \mu_2^{L_2} D^H D}} \tag{S.23}$$

We solved the z_1 subproblem by the soft thresholding operation:

$$\min_{z_1} \lambda_1^{L_2} \left\| z_1 \right\|_1 + \frac{\mu_1^{L_2}}{2} \left\| \nabla \chi_2 - z_1 + s_1 \right\|_2^2 \tag{S.24}$$

$$\boxed{z_1 = \max \left(\left| \nabla \chi_2 + s_1 \right| - \frac{\lambda_1^{L_2}}{\mu_1^{L_2}}, 0 \right) \cdot \operatorname{sign} \left(\nabla \chi_2 + s_1 \right)} \tag{S.25}$$

We solved the z_2 subproblem by the soft thresholding operation as well:

$$\min_{z_2} \left\| \mathbb{W} \cdot z_2 \right\|_2^2 + \frac{\mu_2^{L_2}}{2} \left\| F^H D F \chi_2 - \phi - z_2 + s_2 \right\|_2^2 \tag{S.26}$$

$$\boxed{z_2 = \max \left(\left| F^H D F \chi_2 - \phi + s_2 \right| - \frac{\mathbb{W}}{\mu_2^{L_2}}, 0 \right) \cdot \operatorname{sign} \left(F^H D F \chi_2 - \phi + s_2 \right)} \tag{S.27}$$

The update rules for the Lagrangian multipliers are given by:

$$s_1 \quad = \quad s_1 + F^H E F \chi_2 - z_1 \tag{S.28}$$

$$s_2 \quad = \quad s_2 + F^H D F \chi_2 - \phi - z_2 \tag{S.29}$$

Section C - COSMOS Experiment

	SNR 40			
	L1	L2	L1L2	Ground Truth
Caudate Nuc.	63,5±10,0[12,0]	61,3±9,1[11,0]	62,1±9,3[10,9]	62,3±11,3
Putamen	85,5±13,6[11,7]	83,7±13,2[10,5]	84,6±13,2[10,5]	84,1±15,4
Globus Pallidus	190,2±17,0[9,3]	189,2±18,7[8,1]	189,7±18,7[8,1]	188,7±25,9
Red Nuc.	89,8±14,6[8,7]	85,8±11,6[11,7]	85,1±11,8[12,0]	90,2±16,1
Subs. Nigra	161,7±16,2[10,3]	161,6±19,3[10,1]	161,2±19,5[10,3]	173,4±23,9
Dent. Nuc.	114,5±10,3[8,8]	112,0±9,9[9,3]	111,6±10,0[9,4]	117,6±12,7
Genu (CC)	-15,7±8,5[45,0]	-16,9±5,9[31,5]	-17,3±5,9[30,3]	-21,7±3,6
Splenium (CC)	-12,1±5,8[36,8]	-14,5±5,4[26,0]	-15,0±5,5[24,6]	-16,9±5,0
Capsula Int.	-48,2±8,9[28,6]	-47,5±7,0[23,6]	-47,4±7,0[23,5]	-42,6±8,4
Frontal WM	-14,5±8,6[45,3]	-12,8±7,1[42,8]	-12,5±7,2[43,8]	-15,5±5,0
Occ WM	-29,3±3,0[29,5]	-26,5±4,1[29,2]	-27,6±4,2[28,2]	-29,6±10,1
	SNR 100			
	L1	L2	L1L2	Ground Truth
Caudate Nuc.	64,2±12,3[10,1]	63,7±10,8[8,8]	63,7±11,5[8,9]	62,3±11,3
Putamen	83,6±15,1[7,9]	82,7±14,5[7,1]	84,2±14,3[6,8]	84,1±15,4
Globus Pallidus	189,8±23,8[5,1]	188,2±22,9[4,8]	190,2±23,3[4,8]	188,7±25,9
Red Nuc.	86,9±18,4[8,6]	87,0±15,7[7,8]	86,2±15,7[8,2]	90,2±16,1
Subs. Nigra	168,5±22,7[6,9]	168,5±23,1[6,6]	167,8±23,1[6,7]	173,4±23,9
Dent. Nuc.	114,1±11,2[8,0]	114,1±9,9[7,9]	114,0±9,7[7,8]	117,6±12,7
Genu (CC)	-20,1±5,5[23,0]	-21,9±4,2[19,5]	-21,7±4,2[19,0]	-21,7±3,6
Splenium (CC)	-13,4±7,8[32,8]	-13,0±7,0[30,9]	-14,0±7,6[28,9]	-16,9±5,0
Capsula Int.	-48,7±9,2[19,2]	-50,1±9,0[22,1]	-48,2±8,9[18,7]	-42,6±8,4
Frontal WM	-15,3±4,8[24,3]	-15,0±5,3[29,0]	-15,8±5,3[28,2]	-15,5±5,0
Occ WM	-29,6±7,4[22,3]	-27,2±6,9[25,8]	-28,3±6,8[24,8]	-29,6±10,1
	SNR 300			
	L1	L2	L1L2	Ground Truth
Caudate Nuc.	64,8±12,2[7,6]	65,2±9,8[8,4]	65,2±11,2[7,8]	62,3±11,3
Putamen	83,4±15,0[5,2]	81,8±14,3[6,0]	82,4±13,9[5,7]	84,1±15,4
Globus Pallidus	190,2±25,4[3,0]	188,5±23,5[3,3]	189,7±24,1[3,2]	188,7±25,9
Red Nuc.	86,7±17,1[5,2]	89,7±15,7[4,2]	87,9±16,0[4,7]	90,2±16,1
Subs. Nigra	172,3±24,6[4,0]	173,3±23,6[4,3]	172,8±23,6[4,3]	173,4±23,9
Dent. Nuc.	115,8±10,6[4,6]	116,0±10,1[4,8]	116,1±9,9[4,8]	117,6±12,7
Genu (CC)	-21,1±4,8[16,9]	-20,7±4,1[17,7]	-20,3±3,9[15,6]	-21,7±3,6
Splenium (CC)	-15,7±6,8[20,4]	-12,8±6,6[30,2]	-14,7±6,8[22,8]	-16,9±5,0
Capsula Int.	-46,3±8,1[12,6]	-48,6±7,4[18,2]	-46,6±7,4[13,8]	-42,6±8,4
Frontal WM	-16,2±4,4[15,1]	-16,9±4,2[18,2]	-17,4±4,3[19,0]	-15,5±5,0
Occ WM	-25,8±10,0[17,3]	-24,8±8,9[21,1]	-24,5±9,2[21,2]	-29,6±10,1

Supporting Information Table S1: Local Measurements (Mean Value ± Standard Deviation [Local RMSE], in Parts per Billion) of evalualuation areas for the COSMOS based phantom.

Section D - Challenge 2.0 Experiment

Sim1 NRMSE

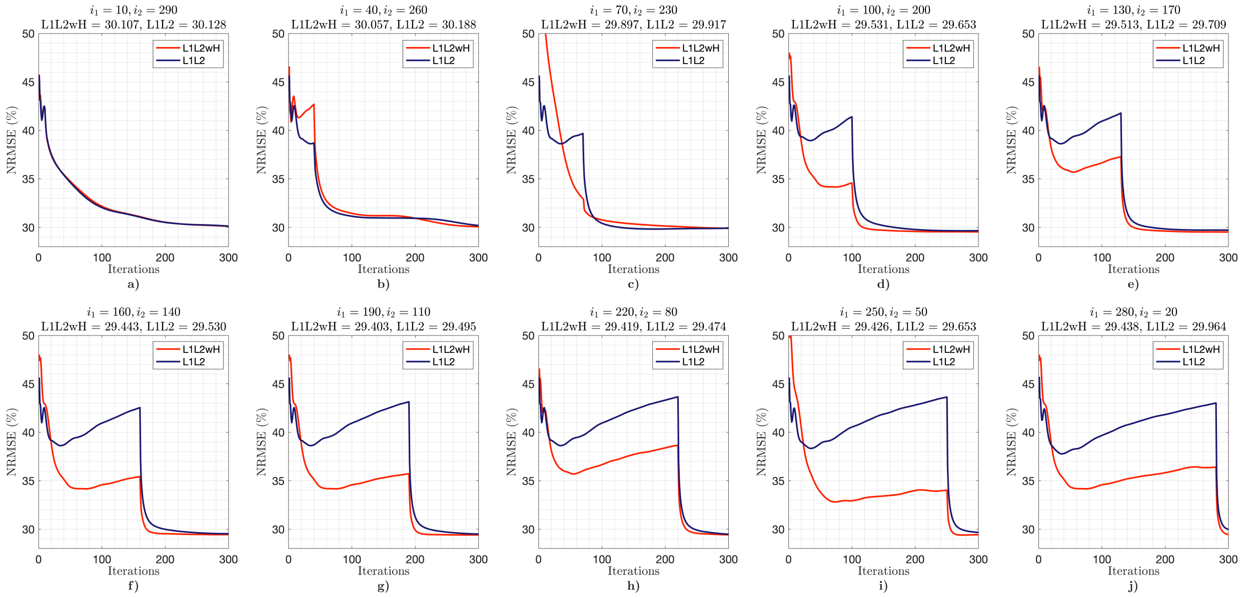

Supporting Information Figure S1: NRMSE evolution of Sim1 optimal reconstructions for different i_1. The error difference obtained between the free parameter method and the proposed heuristic is less than 1 % for all i_1. The difference between the best and the worst reconstruction is less than 1 %, indicating that the number of iterations of L1 norm is not an extremely determinant factor, which confirms the hypothesis that the stage of an L1 norm solution is a better starting point than 0.

Sim2 NRMSE

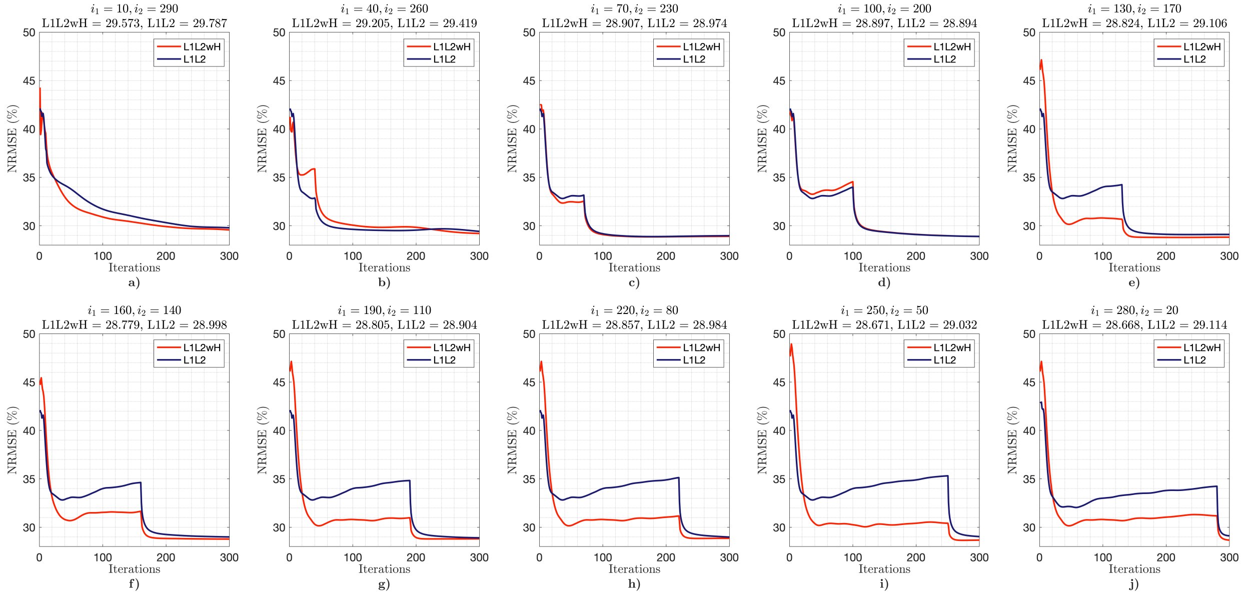

Supporting Information Figure S2: NRMSE evolution of Sim2 optimal reconstructions for different i_1.

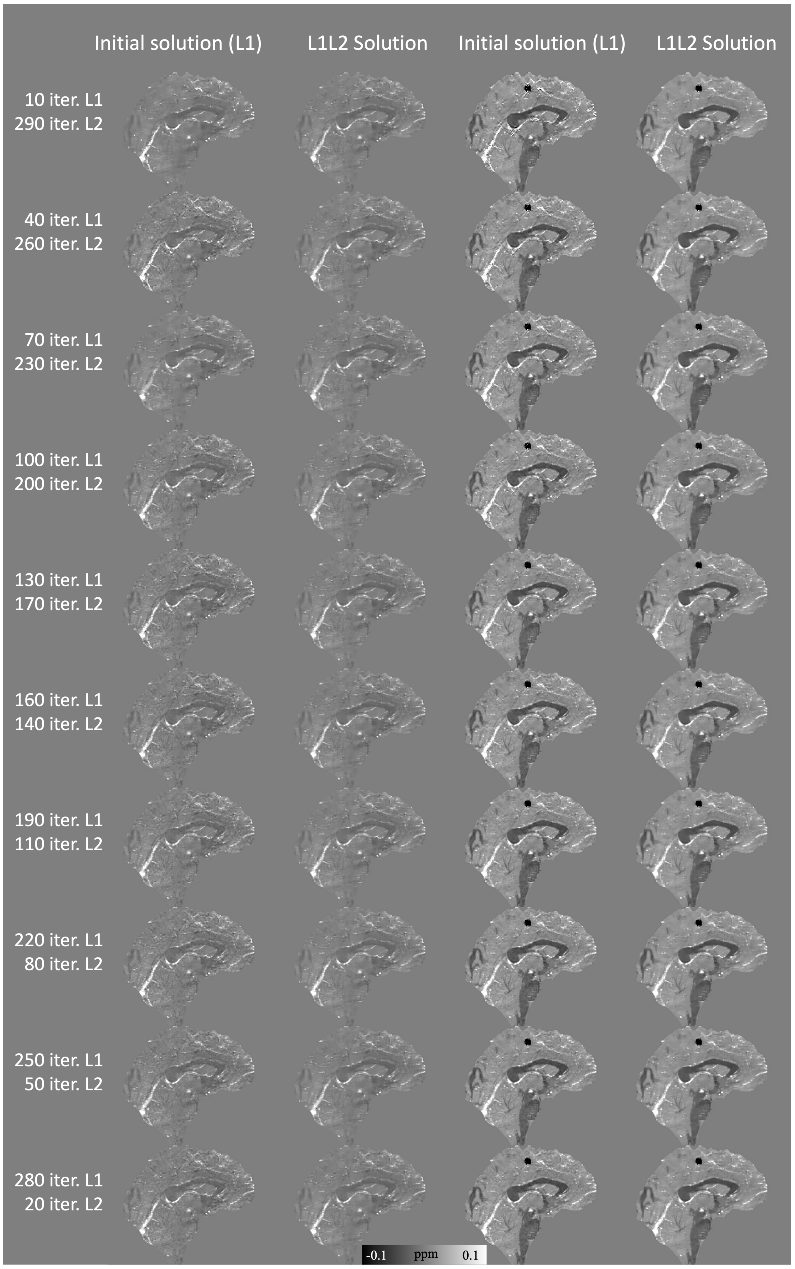

Supporting Information Figure S3: Solutions at the end of stage 1 and stage 2 for Sim1 and Sim2. The solutions at the end of stage 1 show a noisy appearance with streaking artifacts (see around the calcification), but with good structural definition. The final solutions maintain the structural details but do not show the noise and streaking artifacts.

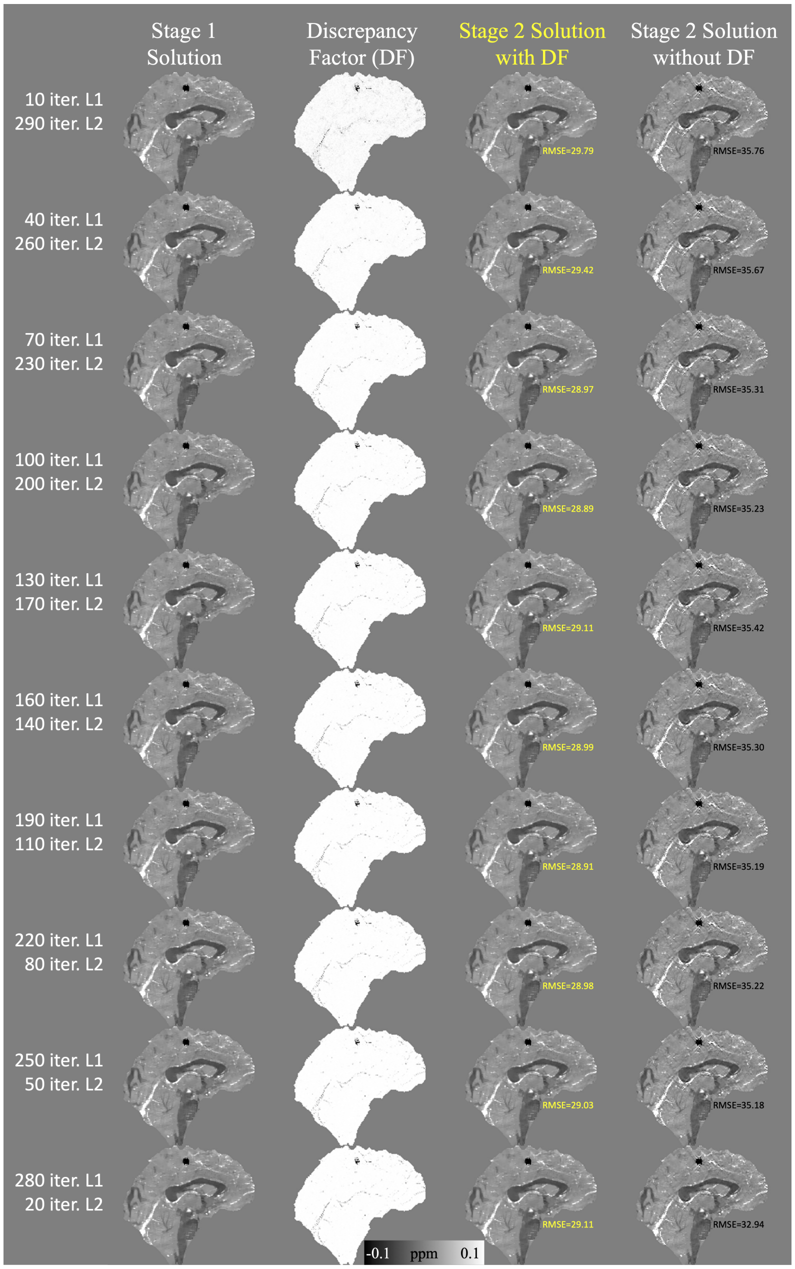

Supporting Information Figure S4: The first column presents the solution at the end of stage 1, the second the discrepancy factor which weights the data consistency weight, the third the solution of stage 2 using the adjustment factor and the fourth the solution without using the adjustment factor with the same parameters.

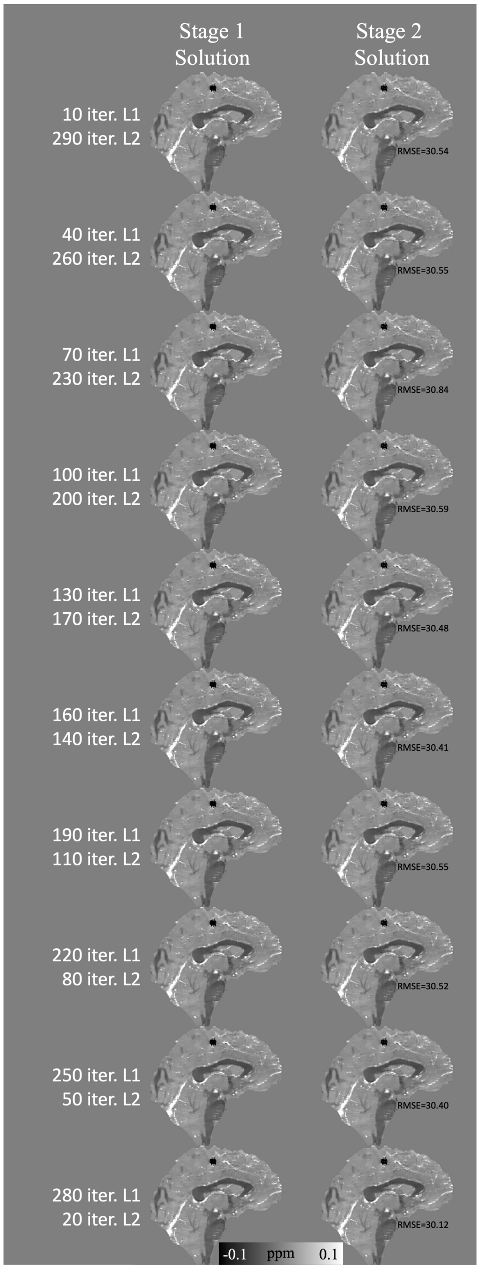

Supporting Information Figure S5: NRMSE optimized solutions of HD-QSM without the discrepancy factor. The first column presents the solution at the end of stage 1 and the second column the final solution.

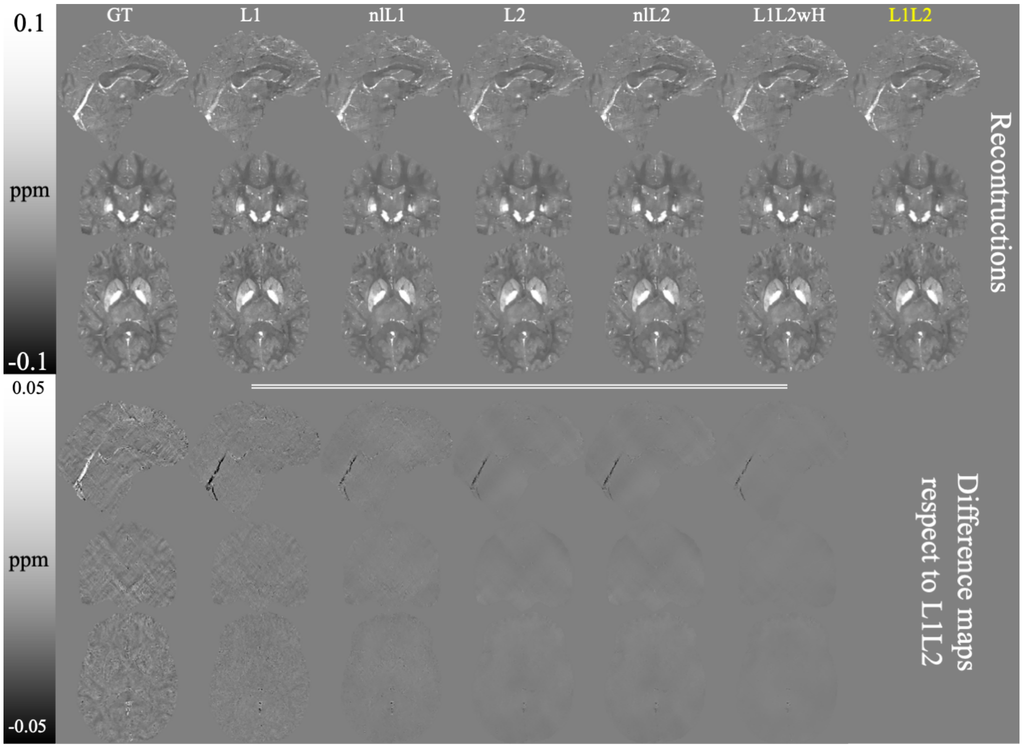

Supporting Information Figure S6: Optimal NRMSE reconstructions of Sim 1. For the search of the optimum of L1L2wH a search was performed in a vector space of $5 \times 5 \times 5 \times 5$ $\left(\lambda_1^{L_1}, \mu_1^{L_1}, \lambda_1^{L_2}, \mu_1^{L_2}\right)$, once the optimum of this space was located, a second search was performed in a space of the same size in the vicinity of the optimum, In total 3 search processes were performed for each simulation, which is 3750 reconstructions, while for L1L2 only 50 reconstructions were necessary.

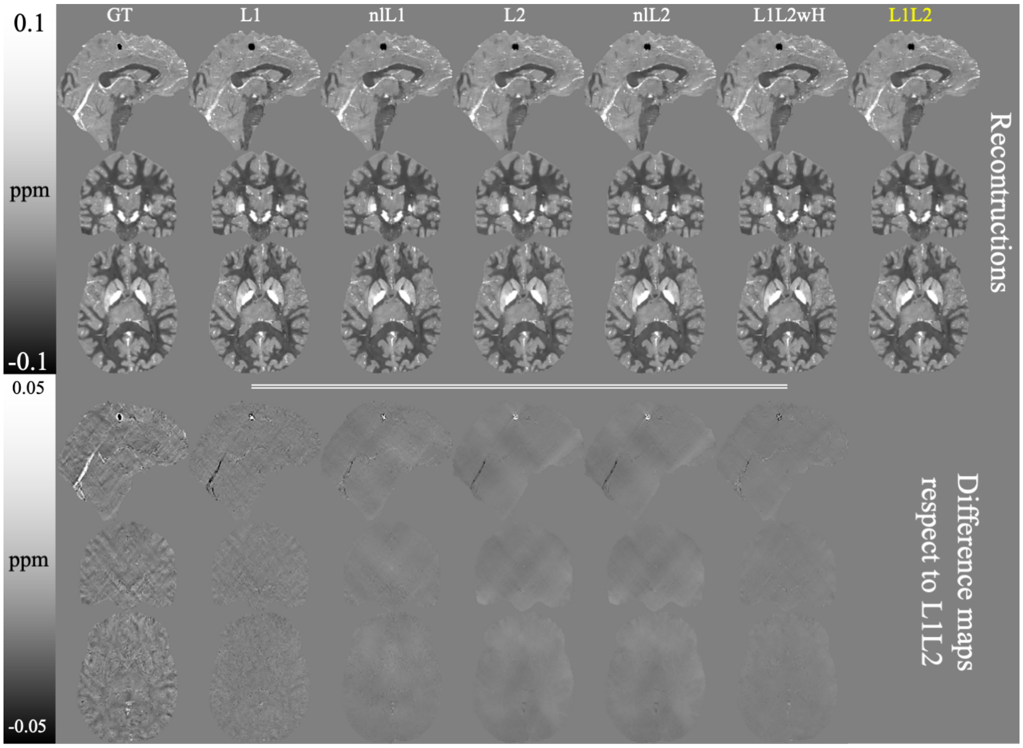

Supporting Information Figure S7: Optimal NRMSE reconstructions of Sim 2. For the search of the optimum of L1L2wH a search was performed in a vector space of $5 \times 5 \times 5 \times 5$ $\left(\lambda_1^{L_1}, \mu_1^{L_1}, \lambda_1^{L_2}, \mu_1^{L_2}\right)$, once the optimum of this space was located, a second search was performed in a space of the same size in the vicinity of the optimum, In total 3 search processes were performed for each simulation, which is 3750 reconstructions, while for L1L2 only 50 reconstructions were necessary.

Section E - Additional In-Vivo Reconstructions

Healthy patient

We performed an in vivo acquisition on a Phillips Ingenia 3T scanner. We used a TGE sequence with five echoes of a healthy patient with the following sequence parameters: TE1=7.2ms, ΔTE=6.2ms, TR=44ms, bandwidth 550.5 Hz and 232×288×64 matrix with 0.59x0.59x1mm^3 voxel size. Phase unwrapping was performed with Laplacian phase unwrapping[40] and background field removal was performed by LBV[41] and VSHARP[40]. We estimated the local field using a magnitude-weighted least-squares phase fitting.

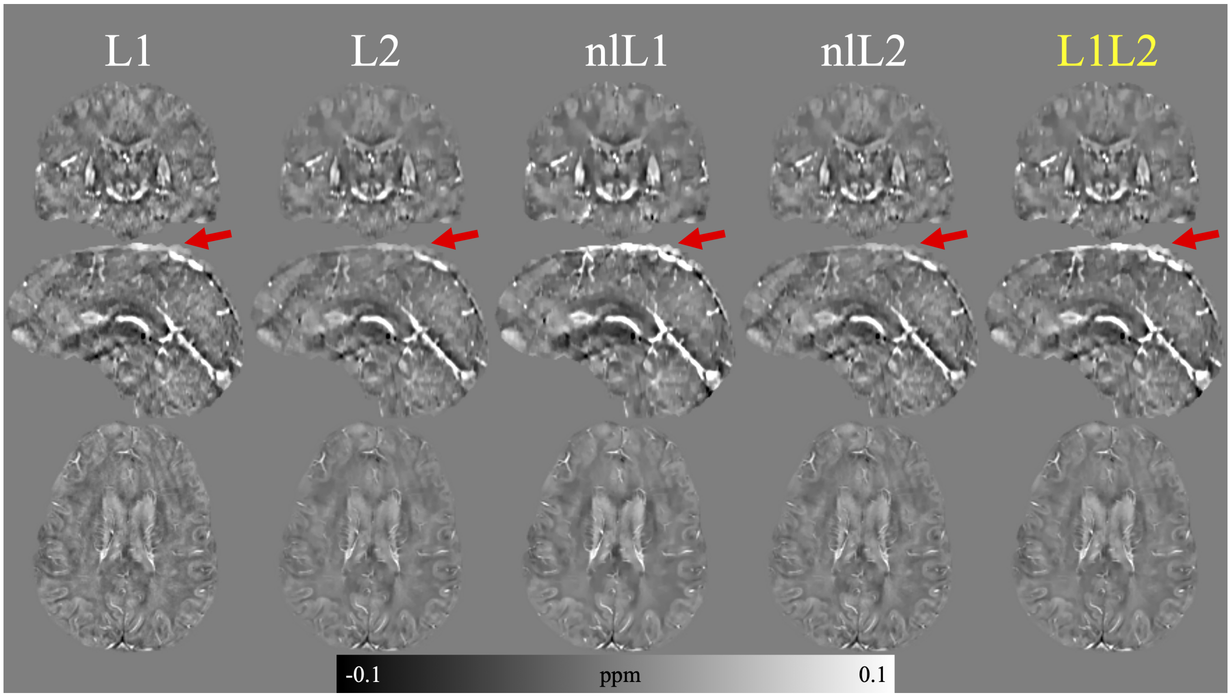

Supporting Information Figure S8: Additional in-vivo reconstruction of healthy patient. The red arrows point to a cortical vein. L1L2 and nlL1 are able to correctly reconstruct the veins of the cortex.

Patient with brain calcification

We performed an in vivo acquisition on a Phillips Ingenia 3T scanner. We used a TGE sequence with five echoes of a healthy patient with the following sequence parameters: TE1=7.2ms, ΔTE=6.2ms, TR=44ms, bandwidth 550.5 Hz and $232\times288\times64$ matrix with 0.59x0.59x1mm^3 voxel size. Phase unwrapping was performed with SEGUE[37] and background field removal was performed by Projection onto Dipole Fields[38]. We estimated the local field using a magnitude-weighted least-squares phase fitting. Background field residuals were removed using the harmonic phase estimation obtained with the Weak-harmonic QSM method (WH-TV)[39]

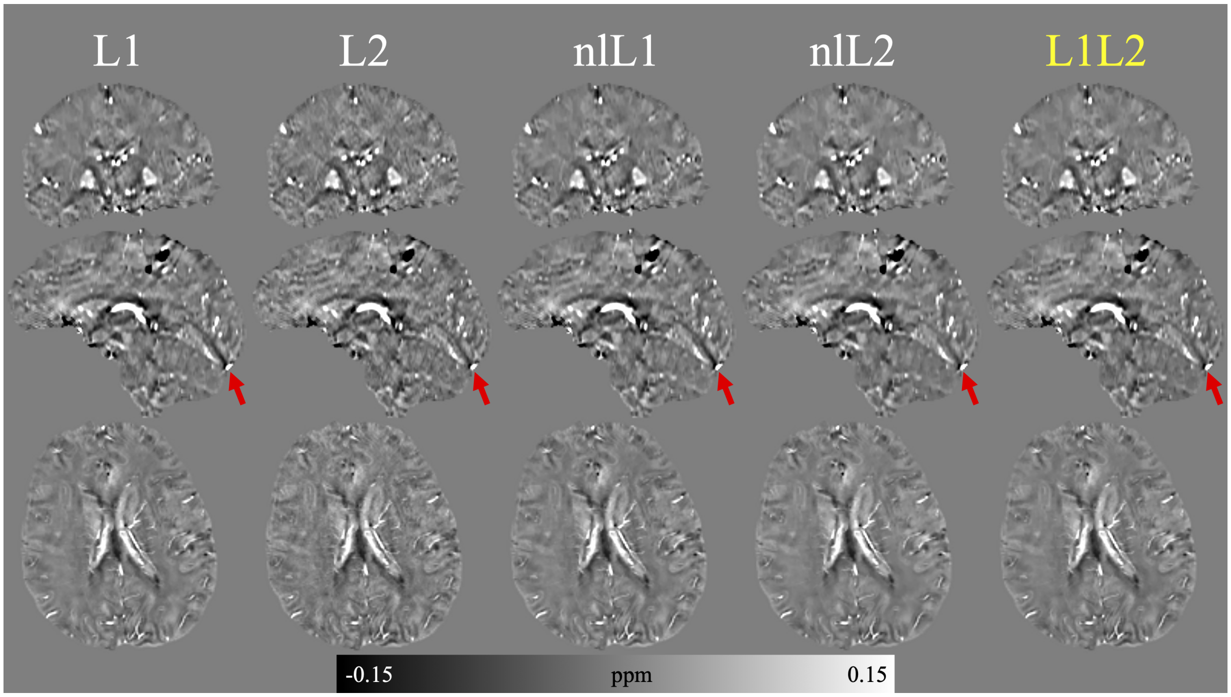

Supporting Information Figure S9: Additional in-vivo reconstruction of a patient with brain calcifications. Red arrows point to the origin of a streaking artifact. The L1L2 reconstruction succeeds in mitigating the spread of this.
